# Expression of the *O*-Glycosylation Enzyme GalNAc-T3 in the Equatorial Segment Correlates with the Quality of Spermatozoa

**DOI:** 10.3390/ijms19102949

**Published:** 2018-09-27

**Authors:** Marie B. Nygaard, Amy S. Herlihy, Charlotte Jeanneau, John E. Nielsen, Eric Paul Bennett, Niels Jørgensen, Henrik Clausen, Ulla Mandel, Ewa Rajpert-De Meyts, Kristian Almstrup

**Affiliations:** 1 Department of Growth and Reproduction, Rigshospitalet, University of Copenhagen, DK-2100 Copenhagen Ø, Denmark; marie.berg.nygaard@regionh.dk (M.B.N.); menisca@gmail.com (A.S.H.); JOHN.ERIK.NIELSEN@regionh.dk (J.E.N.); Niels.Joergensen@regionh.dk (N.J.); Ewa.Rajpert-De.Meyts@regionh.dk (E.R.-D.M.); 2 The Fertility Clinic, Rigshospitalet, University of Copenhagen, DK-2100 Copenhagen Ø, Denmark; 3 Copenhagen Center for Glycomics, Departments of Cellular and Molecular Medicine and Odontology, Faculty of health Sciences, University of Copenhagen, Blegdamsvej 3, DK-2200 Copenhagen N, Denmark; charlotte.jeanneau@univ-amu.fr (C.J.); epb@sund.ku.dk (E.P.B.); hclau@sund.ku.dk (H.C.); ulma@sund.ku.dk (U.M.); 4 International Center for Research and Research Training in Endocrine Disruption of Male Reproduction and Child Health (EDMaRC), Rigshospitalet, University of Copenhagen, DK-2100 Copenhagen Ø, Denmark

**Keywords:** GalNAc-T3, GALNT3, Male fertility, Semen quality, Mucin-type *O*-linked glycosylation, Spermatozoa

## Abstract

We question whether the expression of GalNAc-T3, the only known *O*-GalNAc-transferase present in germ cells, is correlated with qualitative and functional parameters of spermatozoa. We investigated the expression of GalNAc-T3 in ejaculated spermatozoa with immunocytochemistry in swim-up purified and acrosome-reacted spermatozoa from quality-control semen donors and in semen samples from 206 randomly selected men representing a broad spectrum of semen quality. Using donor ejaculates and immunofluorescence detection we found that expression of GalNAc-T3 and the presence of the immature *O*-glycans Tn and T localized to the equatorial segment of spermatozoa. The proportion of GalNAc-T3-positive spermatozoa in the ejaculate increased after swim-up and appeared unaffected by induction of acrosomal exocytosis. The fraction of spermatozoa with equatorial expression of GalNAc-T3 correlated with classical semen parameters (concentration *p* = 9 × 10^−6^, morphology *p* = 7 × 10^−8^, and motility *p* = 1.8 × 10^−5^) and was significantly lower in men with oligoteratoasthenozoospermia (*p* = 0.0048). In conclusion, GalNAc-T3 was highly expressed by motile spermatozoa and the expression correlated positively with the classical semen parameters. Therefore, GalNAc-T3 expression seems related to the quality of the spermatozoa, and we propose that reduced expression of GalNAc-T3 may lead to impaired *O*-glycosylation of proteins and thereby abnormal maturation and reduced functionality of the spermatozoa.

## 1. Introduction

At least in industrialised countries, couples increasingly experience infertility and attend fertility clinics [1]. While male infertility accounts directly for approximately 30% of couple infertility, it is estimated to be a cofactor in an additional 40% of all infertility problems [2]. Analysis of classical semen variables like sperm concentration, total sperm count, motility, and morphology has long been used as predictive markers of fecundity [3,4,5,6]. However, these variables do not describe all aspects of fecundity and are presumably only impaired in a subset of infertile men [7]. Thus, there is still a need for the identification of other factors to understand male fertility potential.

When spermatozoa leave the testes, they are essentially transcriptionally and translationally inert and therefore cannot rely on synthesis of new proteins [8,9,10]. Despite this, the spermatozoa still need to mature in the epididymis; respond to major changes in the environment; find their way in the female reproductive tract by means of rheo-, thermo-, and chemo-taxis; and capacitate, hyperactivate, and undergo acrosomal exocytosis, before they are finally able to bind the plasma membrane and fertilise the egg [11]. All of these functions rely on cellular responses that do not involve protein synthesis and consequently depend on processes such as exchange of ions (e.g., calcium signalling), membrane modulations (e.g., acrosomal exocytosis), and post-translational protein modulations, such as phosphorylation and glycosylation [12].

Glycosylation, the addition of a sugar moiety to a protein, plays an important role in sperm maturation, function, and fertilisation [13]. During spermatogenesis, glycosylation ensures the correct distribution of proteins to their structural and functional compartments, and in the epididymis, glycosylation is involved in the maturation of spermatozoa [14]. After ejaculation, glycoproteins play important roles in the interaction between gametes at the zona pellucida and at the oolemma, ensuring adherence and binding of the two gametes [12,15].

Several different forms of glycosylation exist. The *N*- and mucin-type *O*-glycosylation are the most common. The mucin-type *O*-glycosylation is found in high density on glycoproteins attached or associated with the cell membrane and is known to modulate protein structure, intracellular sorting of proteins, cell-cell adhesion, and cell maturation [16,17,18]. The mucin-type *O*-glycosylation is defined as the covalent attachment of an *N*-Acetylgalactosamine (GalNAc) residue to a polypeptide backbone at the hydroxyl group of serine and threonine. The most immature *O*-glycans are designated Tn (GalNAcα1-*O*-Ser/Thr), sialyl-Tn (NeuAcα2-6GalNAcα1-*O*-Ser/Thr), and T (Galβ1-3GalNAcα1-*O*-Ser/Thr). The majority of these are subsequently elongated and modified to more complex structures with different functions. The initial attachment of GalNAc is catalysed by a large family (20 members) of homologous polypeptide GalNAc-transferases (GalNAc-Ts) and takes place in the Golgi apparatus [18]. The different GalNAc-T isoforms are differentially expressed in different tissues and cells and have different specificities for acceptor peptide sequences [18,19,20]. GalNAc-T1 and -T2 are the most widely distributed GalNAc-Ts, being expressed in most human tissues, whereas GalNAc-T3 has a more restricted expression, e.g., in the testes, kidney, and reproductive and gastrointestinal tracts [21]. We have previously shown that these simple *O*-glycans and the GalNAc-Ts are developmentally regulated in the human testis, and that GalNAc-T3 likely is the only GalNAc-T present in spermatozoa [22]. These findings led to the hypothesis that this restricted glycosylation pattern in germ cells may be crucial for the function of spermatozoa.

We here addressed this hypothesis and investigated the subcellular localisation of GalNAc-T3 and the immediate products the T and Tn *O*-glycans in spermatozoa, and characterised the function of sperm cells with high and low expression of GalNAc-T3 in relation to the acrosome reaction and classical semen parameters.

## 2. Results

### 2.1. GalNAc-T3 Is Expressed in the Equatorial Segment of the Sperm Head and Is Highly Expressed in Motile Sperm Cells

**Figure 1:**
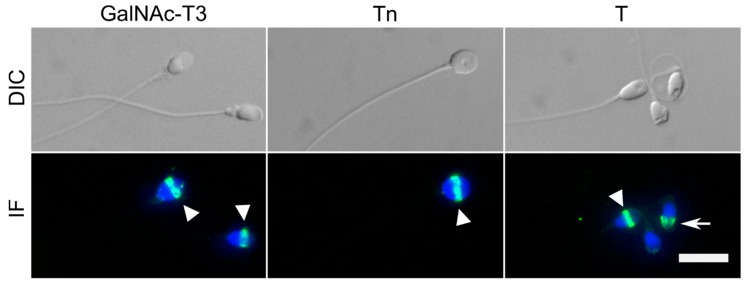
Localization of GalNAc-T3 in human spermatozoa. Top panel: Digital Interference Contrast (DIC) images of human spermatozoa. Lower panel: Immunofluorescence (IF) images of GalNAc-T3 (mAb UH5, 2D10) and simple mucin-type *O*-glycosylation Tn (mAb 5F4) and T (mAb 3C9). Nuclei of the spermatozoa were stained with 4’,6-diamidino-2-phenylindole (DAPI). GalNAc-T3, Tn, and T antigen all localised to the equatorial segment of sperm cells as indicated by the arrowhead. The T antigen was also present in the acrosomal cap (arrow). Scalebar 10 µm.

We detected the expression and localisation of GalNAc-T3 in sperm cells using immunofluorescence. We confirmed that GalNAc-T3 was specifically localised to the equatorial segment of the sperm head (Figure 1), as previously reported [22,23]. We also detected simple *O*-glycosylation patterns in the equatorial segment with antibodies to the short *O*-glycans, T and Tn (Figure 1). The T antigen was also detected in the acrosomal cap (Figure 1).

**Figure 2:**
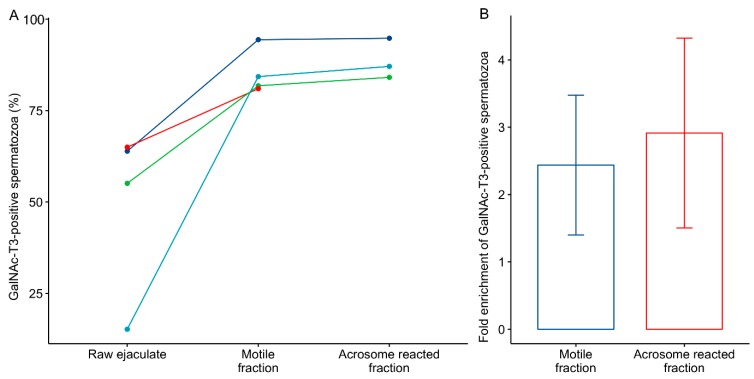

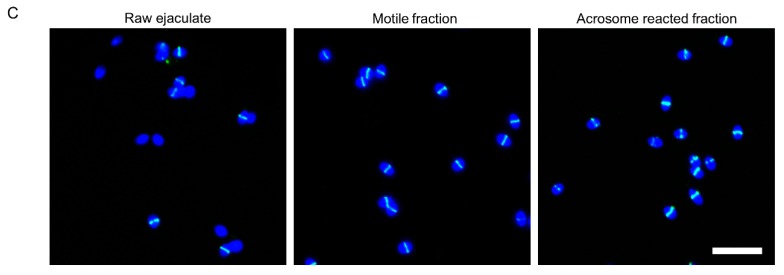
Expression of GalNAc-T3 on spermatozoa in swim-up fractions and after acrosomal exocytosis. (**A**) Ejaculates from 4 laboratory quality control (QC)-donors were evaluated for equatorial GalNAc-T3 expression before and after swim-up selection. Three of the samples were further evaluated after incubation with the ionophore ionomycin, which induces the acrosome reaction. (**B**) Bar plot showing the fold enrichment of GalNAc-T3-positive spermatozoa in the motile and the acrosome-reacted fractions relative to the raw ejaculate. Error bars indicate the standard error of the mean. (**C**) Representative immunofluorescence images of GalNAc-T3 expression detected with mAb 2D10 in the raw ejaculate, the motile fraction and the acrosome-reacted fraction. Nuclei of the spermatozoa were stained with DAPI. Scale bar 20 µm.

GalNAc-T3 was not uniformly expressed in sperm cells in the ejaculate of the donors, and one donor showed only 15% GalNAc-3-positive cells (Figure 2A). The number of spermatozoa that showed equatorial expression of GalNAc-T3 in the ejaculate was significantly (*p* = 0.029) enriched in the motile fraction of spermatozoa purified by the swim-up procedure (*n* = 4; Figure 2A), with a mean fold enrichment of 2.4 (mean 49.8% vs. 85.4%; Figure 2B).

Capacitation and acrosome reaction cause major changes to the membrane composition and protein content, and we therefore further tested whether the equatorial GalNAc-T3 expression was affected by acrosomal exocytosis. Swim-up purified spermatozoa were capacitated and acrosomal exocytosis induced with the calcium ionophore, ionomycin (*n* = 3). Inducing acrosomal exocytosis did not significantly change the fraction of spermatozoa with high equatorial expression of GalNAc-T3 (mean 85.4% vs. 88.7%, Figure 2A,C), and the fold enrichment relative to the raw ejaculate was similar to what was observed for the motile fraction (mean 2.4 vs. 2.9, Figure 2B,C).

### 2.2. Association between GalNAc-T3 Expression and Classical Semen Parameters

To examine if the GalNAc-T3 expression in sperm cells were associated to classical semen parameters like motility, morphology, and concentration, we manually counted the fraction of GalNAc-T3 expressing sperm cells in the ejaculate of the 206 men. We found a very broad spectrum of GalNAc-T3 expression in the ejaculates (0–95% GalNAc-T3 positive sperm cells; Table 1). Approximately 20% (41 men) had less than 10% GalNAc-T3-positive spermatozoa, and 4% (8 men) had more than 90% spermatozoa with equatorial GalNAc-T3 expression.

**Figure 3:**
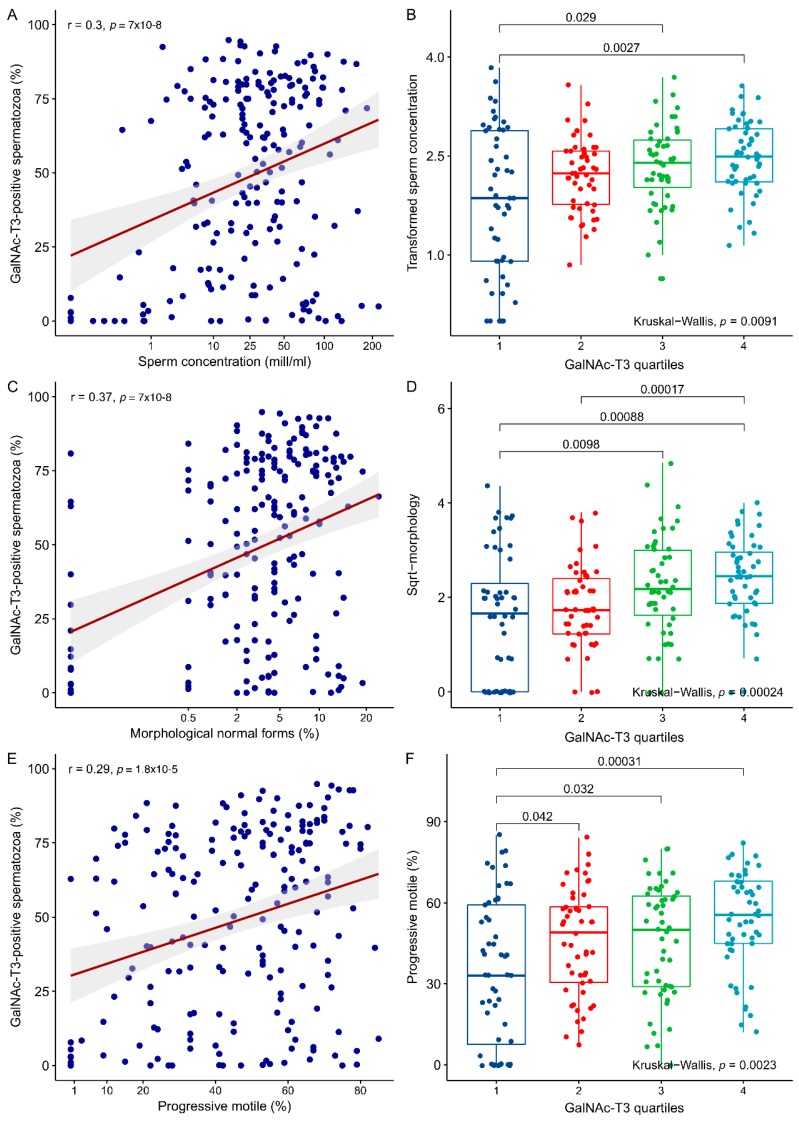
Association between the fraction of GalNAc-T3-positive spermatozoa in the ejaculate and classical semen parameters; sperm concentration, morphology and motility. Scatter plots of the fraction of GalNAc-T3-positive spermatozoa and (**A**) sperm concentration (4th-root transformed), (**C**) the fraction of spermatozoa with normal morphology (square root transformed), and (**E**) the fraction of progressive motile spermatozoa (un-transformed). In each scatter plot, the Pearson correlation quotient (*r*) is indicated together with the *p*-value of the association. The red line indicates the best linear fit and the grey shaded area the confidence interval. The fraction of GalNAc-T3-positive spermatozoa were divided into quartile-bins (1: Lowest and 4: Highest) and plotted against (**B**) sperm concentration (4th root-transformed), (**D**) the fraction of spermatozoa with normal morphology (square root (Sqrt) transformed), and (**F**) the fraction of progressive motile spermatozoa (un-transformed). *p*-values of individual comparisons (Wilcoxon rank sum tests) are indicated on each plot together with the overall significance of the grouping (Kruskal-Wallis test).

The percentage of GalNAc-T3-positive spermatozoa in the ejaculate were significantly associated with sperm concentration (*p* = 9 × 10^−6^), morphology (*p* = 7 × 10^−8^), and progressive motility (*p* = 1.8 × 10^−5^) (Figure 3A,C,E). To further evaluate the effect of GalNAc-T3 expression on semen parameters, we divided the men into quartiles according to the number of GalNAc-T3-positive spermatozoa in the ejaculate (1: <25%, 2: 25–50%, 3: 50–75%, and 4: >75%). This demonstrated differences between the groups of the lowest and highest quartiles of GalNAc-T3 positive spermatozoa and semen parameters (concentration: *p* = 0.0027, morphology: *p* = 8.8 × 10^−4^, motility: *p* = 3.1 × 10^−4^; Figure 3B,D,F). In all cases, a “dose-response relationship” was observed between semen parameters and the quartiles and overall differences, as tested with the Kruskal-Wallis test, also showed significant differences between all groups (concentration: *p =* 0.0091, morphology: *p* = 2.4 × 10^−4^, motility: *p =* 0.0023; Figure 3B,D,F).

**Figure 4:**
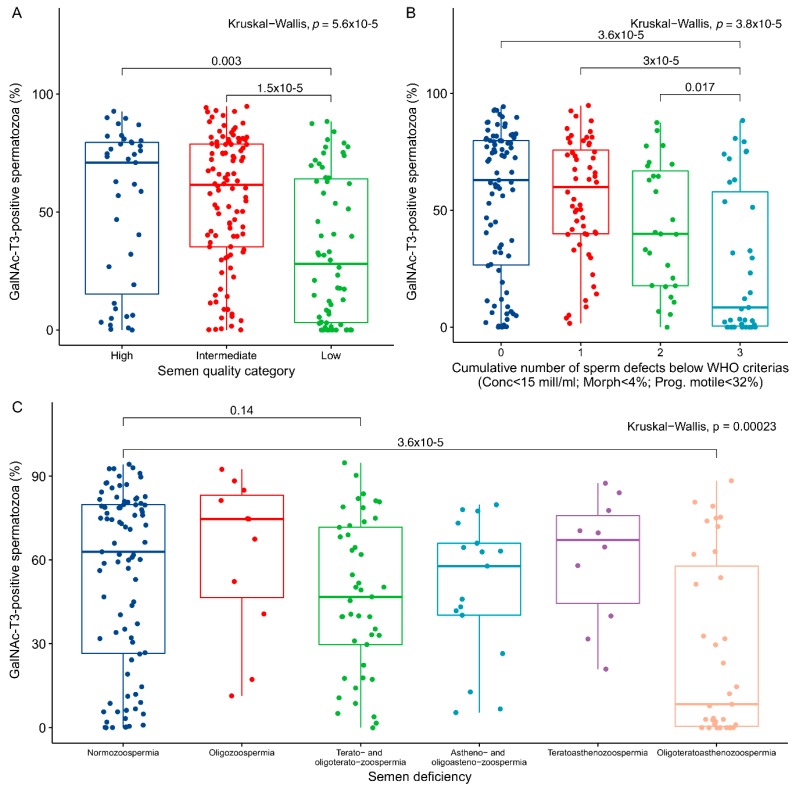
Association between GalNAc-T3 levels and semen quality. (**A**) The samples were divided into categories of high (*n* = 39), intermediate (*n* = 104), and low (*n* = 62) semen quality based on parameters given previously [23] and plotted in a boxplot showing the fraction of GalNAc-T3-positive spermatozoa. (**B**) Boxplot of the fraction of GalNac-T3-positive spermatozoa and the cumulative number of sperm defects as defined by the World Health Organisation (WHO). (**C**) Boxplot of the fraction of GalNAc-T3-positive spermatozoa and the type of semen deficiency observed. *p*-values from comparisons of individual groups by Wilcoxon rank sum tests, as well as the overall significance of the grouping by Kruskal-Wallis tests are indicated on each plot.

To integrate sperm concentration, motility, and morphology into clinically relevant categories, we used the method described previously [24] and divided samples into groups of high (*n* = 39), intermediate (*n* = 104), and low (*n* = 62) semen quality (Figure 4A). A significant difference (*p* = 1.5 × 10^−5^) was observed between men categorised with intermediate and low semen quality together with an overall significance between all the groups (Kruskal-Wallis: *p* = 5.6 × 10^−5^). When samples were classified according to the cumulative number of sperm defects (defined as either a concentration below 15 mill/mL, less than 4% morphological normal forms, or less than 32% motile spermatozoa; [25]), we found a significantly lower number of GalNAc-T3-positive spermatozoa among men with three sperm defects compared to men with no (*p* = 3.6 × 10^−5^), one (*p* = 3 × 10^−5^), or two (*p* = 0.017) sperm defects (Figure 4B). Overall, the number of GalNAc-T3-positive spermatozoa was significantly different between all the groups (Kruskal-Wallis: *p* = 3.8 × 10^−5^). Finally, when men were categorised according to the type of semen deficiency (Figure 4C) we observed differences between normozoospermic men (*n* = 87) and men with oligoteratoasthenozoospermia (*n* = 35; *p* = 3.6 × 10^−5^), and non-significant tendencies of lower GalNAc-T3 levels among men with terato- and oligoteratozoospermia (*n* = 45; *p* = 0.14).

## 3. Discussion

We have previously shown that the pattern of *O*-glycosylation and GalNAc-T isoform expression is tightly regulated during spermatogenesis. The presence of the simple *O*-glycans Tn and T coincided with expression of GalNAc-T3 in spermatocytes and spermatids [22]. Similar patterns were found on ejaculated spermatozoa, albeit with quite some variation between individual spermatozoa. This pattern of expression corresponds also very well to observations in spermatozoa from other species like the bank vole [26]. Here, we have extended our earlier observations by functional studies of sperm with different GalNAc-T3 expression. We have shown that an equatorial expression of GalNAc-T3 is predominantly associated with motile spermatozoa in the swim-up fraction of an ejaculate. This indicates that the fraction of GalNAc-T3 positive spermatozoa are the most motile spermatozoa in the ejaculate and hence, most likely, the spermatozoa of the best quality. Importantly, the fraction of GalNAc-T3 positive spermatozoa did not change when we induced the acrosome reaction in the motile fraction. Induction of acrosomal exocytosis by ionomycin only makes a fraction of the spermatozoa react, but this did not change the observed expression pattern. We have previously shown that the absence of GalNAc-T3 is linked to impaired Tn and T *O*-glycosylation and may be a sign of unsuccessful maturation during spermatogenesis [22]. Taken together, these results indicate that equatorial GalNAc-T3 expression (and the presence of T and Tn *O*-glycosylation) may be related to spermatozoa of good quality.

We further tested the applicability of GalNAc-T3 as a marker for good quality spermatozoa by evaluating the expression in raw ejaculates from men representing a broad spectrum of semen quality. Although a significant association was observed with classical semen parameters, the data also revealed a very large variability. Some men with a low number of GalNAc-T3-positive spermatozoa had a high sperm concentration and many motile and morphologically normal spermatozoa. Nevertheless, when we divided the data into quartiles based on the fraction of GalNAc-T3-positive spermatozoa, we observed a striking gradual increase in association (dose-response like pattern) with all the classical semen parameters. The most significant association was observed with motility and morphology, while the association with sperm concentration was less significant. To further integrate the semen parameters into one combined clinical relevant parameter, we used the guidelines from the World Health Organisation (WHO) on normal levels of semen quality to differentiate men into groups according to the number of sperm defects and categories of high, intermediate, and low semen quality as it has recently been done in other publications [24,27]. This showed that men with a poor semen quality indeed had a significantly lower fraction of GalNAc-T3-positive spermatozoa in their ejaculate. The results, however, indicated that only men with impairment of all classical semen parameters or men in the low-quality group had a significantly lower number of GalNAc-T3-positive spermatozoa. This finding was recapitulated when we analysed the type of sperm defect, which found only oligoteratoastenozoospermic men had significantly lower levels of GalNAc-T3-positive spermatozoa, despite men with impaired morphology (terato- and oligo-teratozoospermic men) also showed lower, but insignificant, levels. Taken together, our data suggests a potential link between *O*-glycosylation patterns and sperm morphology and motility, and thereby potentially proper development of spermatozoa during spermiogenesis and/or epididymal maturation.

As such, the clinical value of the presence of GalNAc-T3 in relation to semen quality seems limited, but an important question is whether this feature is associated with fertility. The equatorial segment of spermatozoa is proposed to be a site of fusion with the egg membrane during mammalian fertilisation [28]. To demonstrate an association with fertility, a larger prospective study that also examines in vitro fertilisation (IVF) outcomes or fertility of the subjects (excluding intracytoplasmic sperm injection (ICSI)) is needed. Oligoteratoastenozoospermic men (equal to men with three cumulative sperm defects) had the lowest number of GalNAc-T3-positive spermatozoa in the ejaculate, which is in line with the phenotype observed in Galnt3 knock-out mice [29]. Teratozoospermic men (together with oligo-teratozoospermic men) nevertheless had the lowest fractions of GalNAc-T3-positive spermatozoa, and sperm morphology showed the best overall significance when quartiles of GalNAc-T3 fractions were analysed. The association between GalNAc-T3 expression and morphology, therefore, seems to be the strongest of the classical semen parameters, and morphology is also the best predictor of pregnancy in IVF [30,31]. Unfortunately, we were not able to test the association between the GalNAc-T3 expression and fertility, because we only had fertility data on a small subset of the men in our study.

Furthermore, the intra-individual variation on successive samples needs to be investigated. Presently, we do not know whether the fraction of GalNAc-T3-positive spermatozoa can vary in time and what factors might influence the fraction positively or negatively. A few men with a complete absence of GalNAc-T3-positive spermatozoa have been identified in this study, and this feature should be further studied. Based on our findings, we speculate that the presence of GalNAc-T3 and *O*-glycosylation patterns might be linked to spermiogenesis within the testes. GalNAc-T3 is present within the Golgi apparatus already in spermatocytes. The Golgi apparatus contributes proteins to the acrosomal vesicle in spermatids, which subsequently forms the acrosome [32]. Aberrations of this traffic could result in a lack of the proper formation or content of the acrosome, including GalNAc-T3. However, it is also possible that epididymal maturation of spermatozoa could affect the GalNAc-T3 expression.

## 4. Materials and Methods

### 4.1. Ethical Considerations

All samples were obtained with informed consent from the patients and controls during their visits at the Department of Growth and Reproduction, section GR-5064, Copenhagen University Hospital (Rigshospitalet) for infertility workup or participation in a research project, respectively (see below). The use of the samples was approved by a local medical research ethics committee (protocol no.: KF 01 273468, 15 August 2005).

### 4.2. Semen Samples

Semen samples were collected from 206 men representing a broad spectrum of semen quality as described before [27]. Samples originated from an unselected and random sub-sample of men from infertile couples (*N* = 125), men from an ongoing study of reproductive health in the general Danish population (*N* = 68), and fertile men (*N* = 13) who previously fathered a child. In addition, semen samples from laboratory QC-donors (*N* = 4) with normozoospermic quality (concentration >15 mill/mL, progressive motile spermatozoa >32%, morphologically normal spermatozoa >4%) were used for the assessment of GalNAc-T3 staining after swim-up and induction of the acrosome reaction.

All participants had been instructed to abstain from ejaculation at least 48 h before delivery of their semen sample. All samples were produced by masturbation, and the ejaculate was collected in wide-mouthed plastic containers. The samples were produced at the Department of Growth and Reproduction, Rigshospitalet, Copenhagen, Denmark in a private room. The samples were incubated at 37 °C for 15–30 min, in order to liquefy, and mixed thoroughly. The semen quality was assessed according to slightly modified WHO procedures as described before [33], and aliquots were drawn for cytospin preparations for GalNac-T3 analysis. Briefly, volume was calculated by weight, and sperm motility was classified as progressive motile (WHO class A+B), non-progressive motile (class C), or immotile (class D). Sperm concentration was assessed using a Bürker-Türk haemocytometer. Only spermatozoa with tails were counted. Smears were made, air dried, fixed in 96% ethanol, and Papanicolaou stained for morphology analysis, according to the strict criteria [34].

### 4.3. Swim-up, Capacitation and Acrosome Reaction

Semen samples from laboratory QC-donors were allowed to liquefy and mixed thoroughly before the motile spermatozoa were purified from the ejaculate by the swim-up technique using human tubular fluid (HTF^+^) medium containing 97.8 mM NaCl, 4.69 mM KCl, 0.2 mM MgSO_4_, 0.37 mM KH_2_PO_4_, 2.04 mM CaCl_2_, 0.33 mM Na-pyruvate, 21.4 mM Na-lactate, 2.78 mM glucose, 21 mM HEPES, and 4 mM NaHCO_3_, adjusted to pH 7.3–7.4 with NaOH, as described elsewhere [27]. After incubation for 1 h at 37 °C, 10% CO_2_ the upper swim-up fraction was carefully collected, and concentration was determined using image cytometry [35,36].

For capacitation, the cell concentration was adjusted to 10 × 10^6^ cells/mL in HTF^+^ medium containing 25 mM NaHCO_3_ and 3 mg/mL (3% [*v*/*v*]) human serum albumin (HSA, Irvine Scientific, Santa Ana, CA, USA) and incubated for at least 3 h at 37 °C in a 10% CO_2_ atmosphere. After capacitation, the sperm cells were induced to undergo acrosome reaction by addition of 2 μM of the calcium ionophore ionomycin (Sigma-Aldrich, St. Louis, MO, USA) and incubated for 30 min at 37 °C at 300 rpm shaking on a thermal mixer [27].

Aliquots were drawn from the raw ejaculate, the motile fraction after swim-up, and the acrosome-reacted sample for cytospins and subsequent immunofluorescence analyses. While all four laboratory QC-donors were evaluated before and after swim-up, only 3 of them were capacitated and acrosome-reacted due to a limited material.

### 4.4. Immunofluorescence (IF)

Mouse monoclonal antibodies (mAbs) raised against human GalNAc-T3 (mAb UH5 (clone 2D10)) and the simple mucin-type *O*-glycans (T (mAb 3C9), Tn (mAb 5F4)) were used in this study. mAb UH5 reacts with the native fold of GalNAc-T3 and does not react by SDS-PAGE Western blotting with the denatured enzyme. The specificity of the GalNAc-T3 mAb has been extensively validated by immunoprecipitation of the active enzyme [37] and by immunocytology using targeted CRISPR/Cas9 knockout of the *GALNT3* gene in human HEK293 cells [38], as well as targeted knock-in of GALNT3 cDNA in human HepG2 cells [39]. mAb UH5 was previously shown to react specifically with human spermatozoa using appropriate mouse IgG1 isotype control mAbs to a panel of other homologous GalNAc-T isoforms [23]. The specificities of mAbs 3C9 and 5F4 have been extensively characterised by ELISA with pure glycoproteins and glycolipids [40,41] and more recently by immunocytology with a panel of human cell lines with ZFN and CRISPR/Cas9 knockout of the core1 synthase chaperone *COSMC* [42,43]. Batches of hybridoma supernatants for each mAb were, in addition, tested by immunocytology with genetically engineered cell lines for performance and specificity before use.

Cytospins of semen were air-dried before fixation in 100% ice-cold acetone (10 min) or 10% formalin (15 min at 4 °C) and permeabilisation in 0.1% Triton-X100 for 10 min at room temperature followed by a wash in Tris-buffered saline (TBS). Unspecific binding was blocked using 10% goat serum (Thermo Scientific, Histostain kit, code 956543B, Waltham, MA, USA) or 5% bovine serum albumin (BSA) for 30 min, and the cells were incubated with primary antibodies (undiluted hybridoma culture supernatants containing 10–20 µg/mL) overnight at 4 °C in a humidity chamber. Negative control antibodies included isotype and subclass IgG1 and IgM mAbs to another GalNAc-T2 isoform (UH4-4C4, IgG1) not expressed in sperm, and to histo-blood group A and H carbohydrate antigens not expressed (HH5 (IgM), HH14 (IgM)) [22]. The cytospins were washed thoroughly for excess primary antibody before incubation with a fluorochrome-conjugated secondary antibody specific for IgG (R37114, Thermo Scientific, Waltham, MA, USA) or IgM (A-21043, Thermo Scientific, Waltham, MA, USA) for one hour at room temperature. The nuclei were stained with DAPI before mounting with antifade mounting medium (Prolong^TM^ Gold Antifade mountant, #P36930, Termo Fisher Scientific, Waltham, MA, USA).

The samples were inspected with an Olympus BX-61 microscope (Olympus BX61, Olympus, Ballerup, Denmark) and images captured using the Cell Sense Dimensions V1.6 software (Olympus Ltd., Ballerup, Denmark). Further image processing was performed with FIJI ImageJ 1.49.

### 4.5. Immunocytochemistry (ICC)

Cytospins were fixated in 100% ice-cold acetone (10 min) and briefly rinsed in double-distilled water (ddH_2_O). Cells were washed in TBS and blocked in 10% goat serum (Thermo Scientific, Histostain kit, code 956543B, Waltham, MA, USA) for 30 min at room temperature. The cytospins were incubated with undiluted mouse monoclonal anti-GalNAc-T3 overnight at 4 °C in a humidity chamber. Excess antibody was removed by a wash in TBS before incubation with biotinylated goat anti-mouse antibody (Thermo Scientific, Histostain kit, code 956543B) for 30 min. The cytospins were washed in TBS and incubated with peroxidase-conjugated Streptavidin complex (Thermo Scientific, Histostain kit, code 956543B) for 30 min. Visualization was performed with aminoethyl carbasole (AEC) (Thermo Scientific, Histostain kit, code 956543B), and cells were counterstained with Meyer’s haematoxylin.

### 4.6. Evaluation of GalNAc-T3 Staining

To reduce sampling error, at least 200 sperm cells from a representative field of views on each cytospin were evaluated for staining of GalNAc-T3. Only sperm cells (counted by DAPI nuclei staining) that had a clearly defined staining along the equatorial segment where counted as positive. Evaluations were performed in two different periods and by two different investigators (J.E.N. and A.H.) by two different methods (ICC and IF). There was no difference in GalNAc-T3 evaluation (*p =* 0.13) between the two different methods (and investigators), when a subset of samples from men with a good semen quality (sperm concentration >50 mill/mL, >40% progressive motile, and >10% morphological normal forms) were analysed, indicating that no systematic differences existed between these two types of evaluation.

### 4.7. Statistical Analysis

The data was analysed in the statistical software program R [44]. Descriptive statistics were obtained from the fBasics function on untransformed values. Some semen parameters were not normal distributed and were therefore transformed to achieve a better approximation of normality (Figure S1). Sperm concentration was transformation using the 4-th root and morphology the square root, which in both cases reduced the kurtosis and skewness (Figure S1). Motility was not transformed as the kurtosis and skewness was already low and was not reduced with different kinds of transformations (Figure S1). Linear regression was used to test for association between percentages of GalNAc-T3 positive sperm cells and semen parameters. The Pearson correlation quotient (*r*) and *p*-values are given on scatter plots. Based on the fraction of GalNAc-T3 expressing spermatozoa, samples were divided into groups representing each GalNAc-T3 quartile. A Wilcoxon Rank Sum test was used to calculate significant differences between GalNAc-T3 quartiles and semen parameters as well as between groups of different semen quality. Overall differences between groups were tested with the Kruskal-Wallis test. All plots were produced with the *ggpubr* package in R. *p*-values below 0.05 were considered significant.

## 5. Conclusions

We confirmed that the simple *O*-glycans T and Tn and GalNAc-T3 were present specifically in the equatorial segment of spermatozoa. Spermatozoa with an equatorial GalNAc-T3 expression were highly enriched in swim-up fractions of ejaculates; hence the GalNAc-T3 expression seems to mark spermatozoa of good quality. The number of GalNAc-T3-positive spermatozoa correlated with classical semen parameters. Men with poor semen quality, and in particular men with oligoteratoasthenozoospermia, had a lower fraction of GalNAc-T3-positive spermatozoa in the ejaculate. Based on our results we suggest that GalNAc-T3 expression is related to the quality of spermatozoa and future research should deduce whether GalNAc-T3 expression holds any predictive value in relation to fertility.

## Figures and Tables

**Table 1:** Descriptive statistics of the included samples.

Semen Parameter	*N*	Mean	Median	Min	Q_1_	Q_3_	Max
Volume (mL)	206	3.8	3.7	0.9	2.8	4.7	7.4
Concentration (mill/mL)	206	39.7	28.0	0.0	9.6	58.0	217.0
Total count (mill)	206	148.8	106.1	0.0	37.6	221.5	1215.2
Progressive motile (%)	206	44.9	48.0	0.0	28.0	63.0	85.0
Morphological normal forms (%)	205	5.3	4.5	0.0	2.0	8.0	23.5
GalNAc-T3 positive spermatozoa (%)	206	48.4	55.5	0.0	17.4	76.5	94.8

## Supplementary Materials

Supplementary materials can be found at http://www.mdpi.com/1422-0067/19/10/2949/s1.
