# Supplementary figures and images for: Expression of the O-Glycosylation Enzyme GalNAc-T3 in the Equatorial Segment Correlates with the Quality of Spermatozoa
Source: Int J Mol Sci. 2018 Sep 27;19(10):2949. doi: 10.3390/ijms19102949 (PMC6212898; doi:10.3390/ijms19102949)

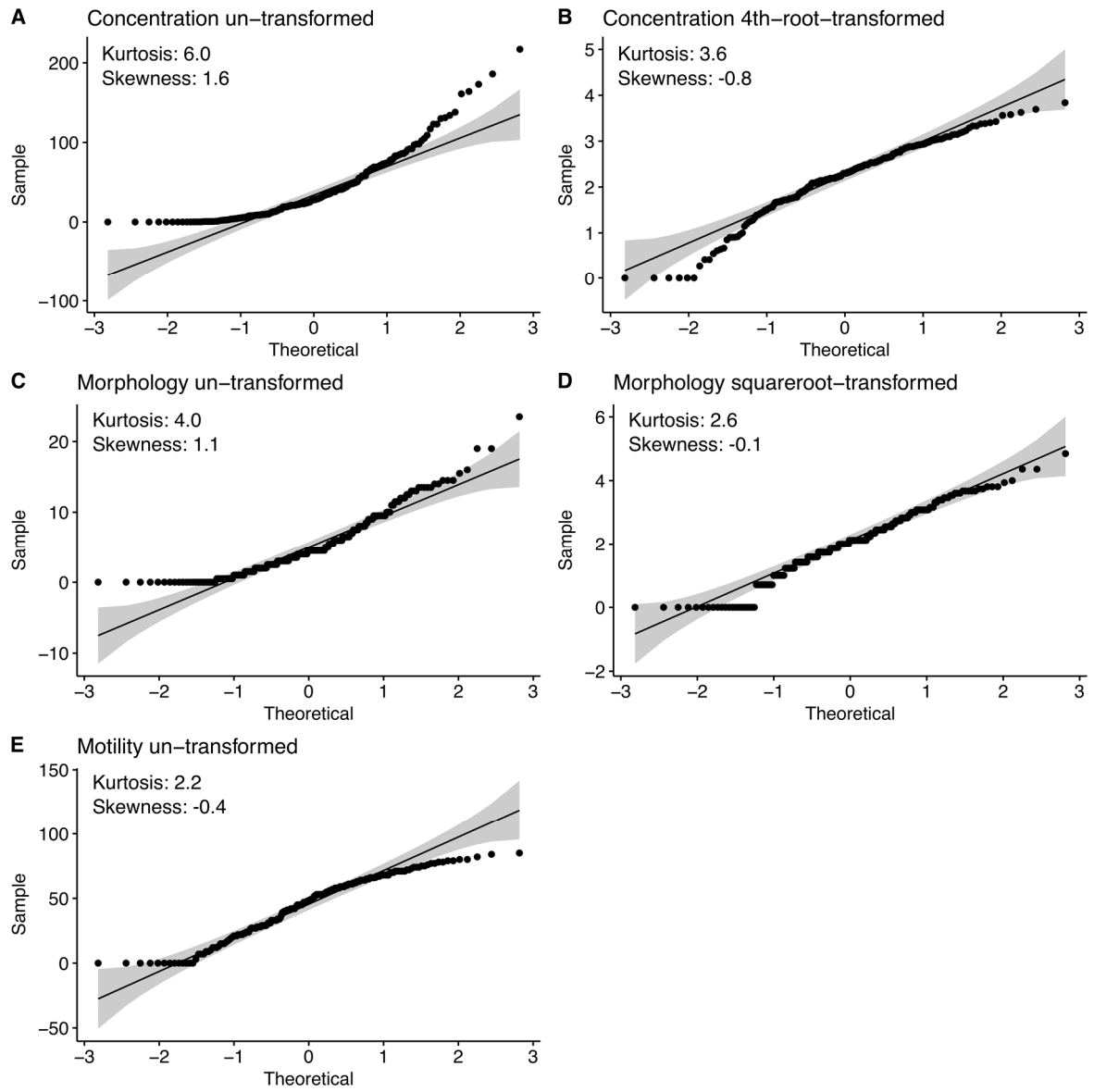

Supplement: Supplementary file 1 [file ijms-19-02949-s001.pdf]
